# Associations between low serum levels of ANRIL and some common gene SNPs in Iranian patients with premature coronary artery disease

**DOI:** 10.1038/s41598-024-51715-2

**Published:** 2024-01-13

**Authors:** Elham Taheri Bajgan, Ali Zahedmehr, Farshad Shakerian, Majid Maleki, Hooman Bakhshandeh, Seyed Javad Mowla, Mahshid Malakootian

**Affiliations:** 1 Molecular Genetics Department, Faculty of Biological Sciences, Tarbiat Modares University, Tehran, Iran; 2 Cardiovascular Intervention Research Center, Rajaie Cardiovascular Medical and Research Center, Iran University of Medical Sciences, Tehran, Iran; 3 Cardiogenetic Research Center, Rajaie Cardiovascular Medical and Research Center, Iran University of Medical Sciences, Tehran, Iran

**Keywords:** Gene expression analysis, Genetic techniques, Genomic analysis, Isolation, separation and purification, Sequencing, Gene expression, Genetic association study, Genetic linkage study, Genetic markers, Genotype, Medical genetics

## Abstract

Coronary artery disease (CAD) is the major cause of mortality in the world. Premature development of CAD can be attributed to women under 55 and men under 45. Many genetic factors play a part in premature CAD. Among them, *ANRIL*, a long noncoding RNA is located at the 9p21 risk locus, and its expression seems to be correlated with CAD. In the current study, premature CAD and control blood samples, with and without Type 2 Diabetes (T2D), were genotyped for six SNPs at the 9p21 locus. Additionally, *ANRIL* serum expression was assessed in both groups using real-time PCR. It was performed using different primers targeting exons 1, 5–6, and 19. The χ^2^ test for association, along with t-tests and ANOVA, was employed for statistical analysis. In this study, we did not find any significant correlation between premature coronary artery disease and rs10757274, rs2383206, rs2383207, rs496892, rs10757278 and rs10738605. However, a lower *ANRIL* expression was correlated with each SNP risk genotype. Despite the correlation between lower *ANRIL* expression and CAD, Type 2 diabetes was associated with higher *ANRIL* expression. Altogether, the correlation between *ANRIL* expression and the genotypes of the studied SNPs indicated that genetic variants, even those in intronic regions, affect long noncoding RNA expression levels. In conclusion, we recommend combining genetic variants with expression analysis when developing screening strategies for families with premature CAD. To prevent the devastating outcomes of CAD in young adults, it is crucial to discover noninvasive genetic-based screening tests.

## Introduction

Coronary artery disease (CAD) is the leading cause of mortality around the globe. In developed and developing countries, CAD is the foremost cause of death. According to a previous study, people in the Middle East demonstrate heart disease at younger ages^1^. In Iran, 50% of annual deaths are the consequence of CAD^2^.

CAD usually affects people aged above 50 years. Nonetheless, in some cases, women under 55 and men under 45 years develop CAD, termed “premature CAD”^3^. No universally accepted age threshold exists for premature CAD. A prior investigation assigned the age of 49 as the cutoff for premature CAD in males based on several autopsy reports^4^. Here, we determined the age of 49 as a cutoff for assigning premature CAD in males.

Patients with premature CAD tend to develop consequent ischemic events at a higher rate than other age groups^5^.CAD is the consequence of atherosclerosis, an inflammatory disease contributing to the accumulation of fatty deposits within the arterial wall which leads to atherosclerotic plaque formation^6^.

The results of a recent genome-wide association study (GWAS) ascribed many loci as risk loci for premature CAD. Among these, the 9p21.3 locus is the most striking genetic risk factor. The 58-kb region of the 9p21 locus is assigned as the CAD risk interval region. No protein-coding gene resides in this segment. The risk haplotype in this region includes interlinked noncoding single-nucleotide polymorphisms (SNPs)^7^.

*CDKN2B-AS1*, also known as “*ANRIL* (antisense noncoding RNA in the *INK4A* locus)”, is a long noncoding RNA (lncRNA) overlapping the *CDKN2B* gene. Further, rs10757274 (NC_000009.12:g.22096056A>G), rs2383206 (g.22115027A>G), rs2383207 (g.22115960A>G), rs496892 (g.22024352C>T), rs10757278 (g.22124478A>G), and rs10738605 (g.22049131C>G) reside in the 9p21 locus. While rs10757274, rs2383206, rs2383207, and rs496892 are in the intronic regions of the *ANRIL* sequence, rs10738605 resides in exon six of *ANRIL* (isoform. 1). On the other hand, rs10757278 is in the intergenic region downstream of the *ANRIL* gene. Extensive GWAS analyses have been conducted on the associations between the abovementioned SNPs and CAD^8,9^. In our study, however, we narrowed down the CAD-associated GWAS variants to SNPs that appeared in publications most frequently as CAD-associated variants in different populations.

Of the 14 splicing isoforms of *ANRIL* annotated on Ref Seq, five isoforms are short and nine are long. The expression pattern of the two groups differs noticeably. The expression of short isoforms is higher than that of long ones. Specific genetic variants are associated with increased levels of short variants and a reduced expression of long isoforms suggesting the contribution of genetic variants to *ANRIL* expression^9,10^.

A prior study discovered that the relatively lower *ANRIL* expression could be increased following treatment^11^. The interplay between the genotypes of 9p21 risk SNPs and *ANRIL* expression has been extensively explained, with research having validated differential exon expression levels of *ANRIL*, along with various circular and linear isoforms^10,12–14^.

In vascular smooth muscle cells and mononuclear cells, *ANRIL* promotes proliferation and atherosclerosis progression^12^. It has been demonstrated that *ANRIL* is upregulated in serum samples from patients with multiple malignancies, including glioma and breast cancer^15,16^. The higher expression of circulating *ANRIL* in the peripheral blood and serum of patients with diabetes and ischemic stroke has been reported^17,18^. As demonstrated by Holdt and Teupser^12^, atherosclerosis progression is associated with decreased expression of circular *ANRIL* and increased expression of linear *ANRIL*.

Circulating transcripts are enriched in serum compared to whole blood sharing 80% of RNAs with other tissues^19^. Serum is more sensitive than plasma in biomarker discoveries^20^ and being free of EDTA, which induces platelet activation, provides a more realistic picture of circulating RNA's physiological pathways^21^.

As proposed by Lawford, from the therapeutic intervention perspective, the earlier the diagnosis is established, the better the outcome will be^6^. Early diagnosis of CAD is crucial in Iran due to the increasing incidence of premature CAD in recent years. Sedentary lifestyles, familial CAD, type 2 diabetes (T2D), hyperlipidemia, and smoking are the most frequently correlated risk factors, although the precise involvement of genetic susceptibility factors should not be underestimated in population-based studies^22,23^.

In the present study, we aimed to investigate the associations between the genotypes of six SNPs within (rs10757274, rs2383206, rs2383207, rs496892, and rs10738605) and proximal to (rs10757278) the *ANRIL* gene sequence, in addition to *ANRIL* expression, and PCAD solely and in combination. According to similar studies in other populations, we hypothesized that circulating *ANRIL* expression differs between premature CAD and non-CAD groups. We also assumed that in premature CAD patients of the Iranian population, a correlation exists between the genetic profile of *ANRIL* SNPs, rs10757278 and *ANRIL* expression, since it has been proposed that genetic factors are more important than environmental factors in the development of CAD among young adults. The growing number of premature CAD occurrences in Iran prompted us to investigate the reasons.

## Results

### Clinical and demographic features represented an association with premature CAD

The results of clinical and demographic characteristic analysis are included in Table 1. The levels of triglyceride, total cholesterol, low-density lipoprotein, and fasting blood sugar were significantly associated with premature CAD. Furthermore, T2D, stroke history, and familial CAD were significantly more frequent in the premature CAD group than in the non-CAD group. The distribution of age in CAD and non-CAD groups respective to and irrespective of sex is depicted in S1 Figure.

**Table 1:** Clinical and demographic characteristics of the included patients.

Parameters	CAD (n = 93)	Non-CAD (n = 87)	*P*-value	*P*-value summary
Age (years), (mean ± SD)				
Female	45.07 ± 3.738	41.82 ± 7.920	0.0655†	ns
Male	39.98 ± 5.034	36.93 ± 5.497	0.0569**†**	ns
BMI (kg/m^2^), (mean ± SD)	28.52 ± 4.525	27.93 ± 5.999	0.2177	ns
SBP (mean ± SD)	127.0 ± 13.71	124.9 ± 11.95	0.3890	ns
DBP (mean ± SD)	78.64 ± 6.372	79.22 ± 5.520	0.8449	ns
Triglyceride (mg/dL), (mean ± SD)	157.3 ± 93.87	116.6 ± 54.86	0.0033	** (P < 0.05)
TC (mg/dL), (mean ± SD)	148.4 ± 47.31	132.5 ± 30.18	0.0153	* (P < 0.05)
HDL-C (mg/dL), (mean ± SD)	36.10 ± 7.177	38.19 ± 7.776	0.753	ns
LDL-C (mg/dL), (mean ± SD)	83.77 ± 37.07	72.39 ± 21.76	0.0207	* (P < 0.05)
FBS (mg/dL), (mean ± SD)	136.3 ± 52.58	101.5 ± 30.11	< 0.0001	**** (P < 0.05)
Stroke history (% frequency)	28.26%	7.93%	0.0019	** (P < 0.05)
T2D (% frequency)	34.78%	12.69%	0.0025	** (P < 0.05)
Familial CAD (% frequency)	78.49%	62.5%	0.0316	* (P < 0.05)
Familial BP (% frequency)	68.47%	73.77%	0.5872	ns
Familial T2D (% frequency)	48.38%	40.32%	0.4103	ns
Familial high cholesterol (% frequency)	47.82%	37.09%	0.2460	ns
Familial MI (% frequency)	59.13%	49.18%	0.2489	ns
Smoking (% frequency)	36.55%	24.28%	0.1244	ns
Alcohol drinking (% frequency)	19.56%	12.90%	0.3808	ns
Stress (% frequency)	75.26%	83.07%	0.3254	ns

*BMI* body mass index, *CAD* Coronary Artery Disease, *DBP* diastolic blood pressure, *FBS* fasting blood sugar, *HDL-C* high-density lipoprotein-cholesterol, *LDL-C* low-density lipoprotein-cholesterol, *ns* not significant, *SBP* systolic blood pressure, *T2D* Type 2 diabetes, *TC* total cholesterol.

^a^†*P*-value for the comparison of age between CAD and Non-CAD irrespective of sex = 0.0745 (ns).

### *ANRIL* was expressed differentially in the premature CAD group compared with the non-CAD group

**Figure 1:**
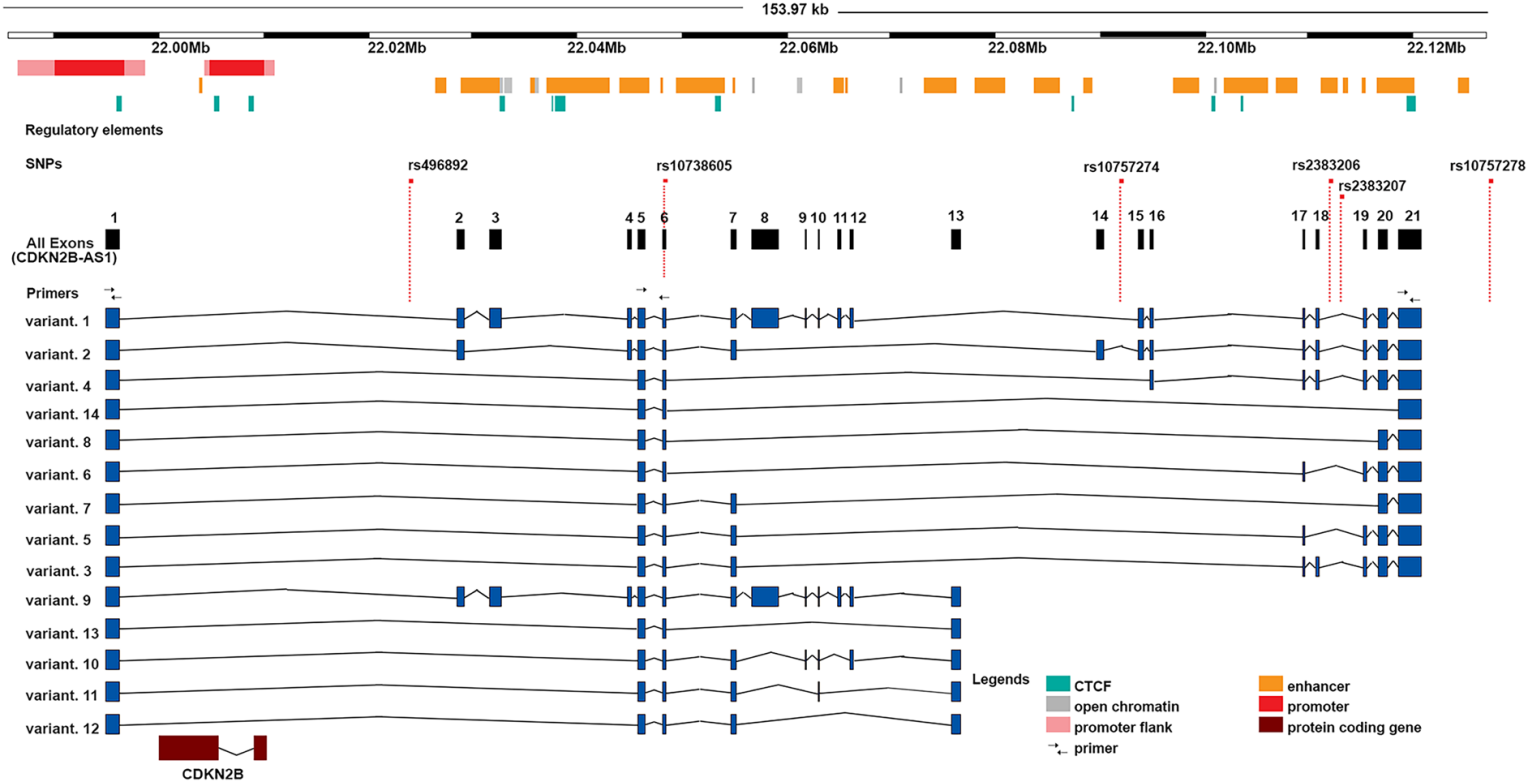
Genomic location of *ANRIL* (*CDKN2B-AS1*) and its splicing variants. *ANRIL* resides in 9p21 locus and spans approximately 126 kb of genomic DNA, comprising 20 exons in total (black rectangles) and 14 splicing isoforms annotated in RefSeq records. The main regulatory elements are depicted below the scale bar. The discussed SNPs are indicated above the *ANRIL* exons. The orientations of primers are displayed by arrows above the exons. *ANRIL*, antisense noncoding RNA in the *INK4A* locus. *CTCF* CCCTC-binding factor.

**Figure 2:**
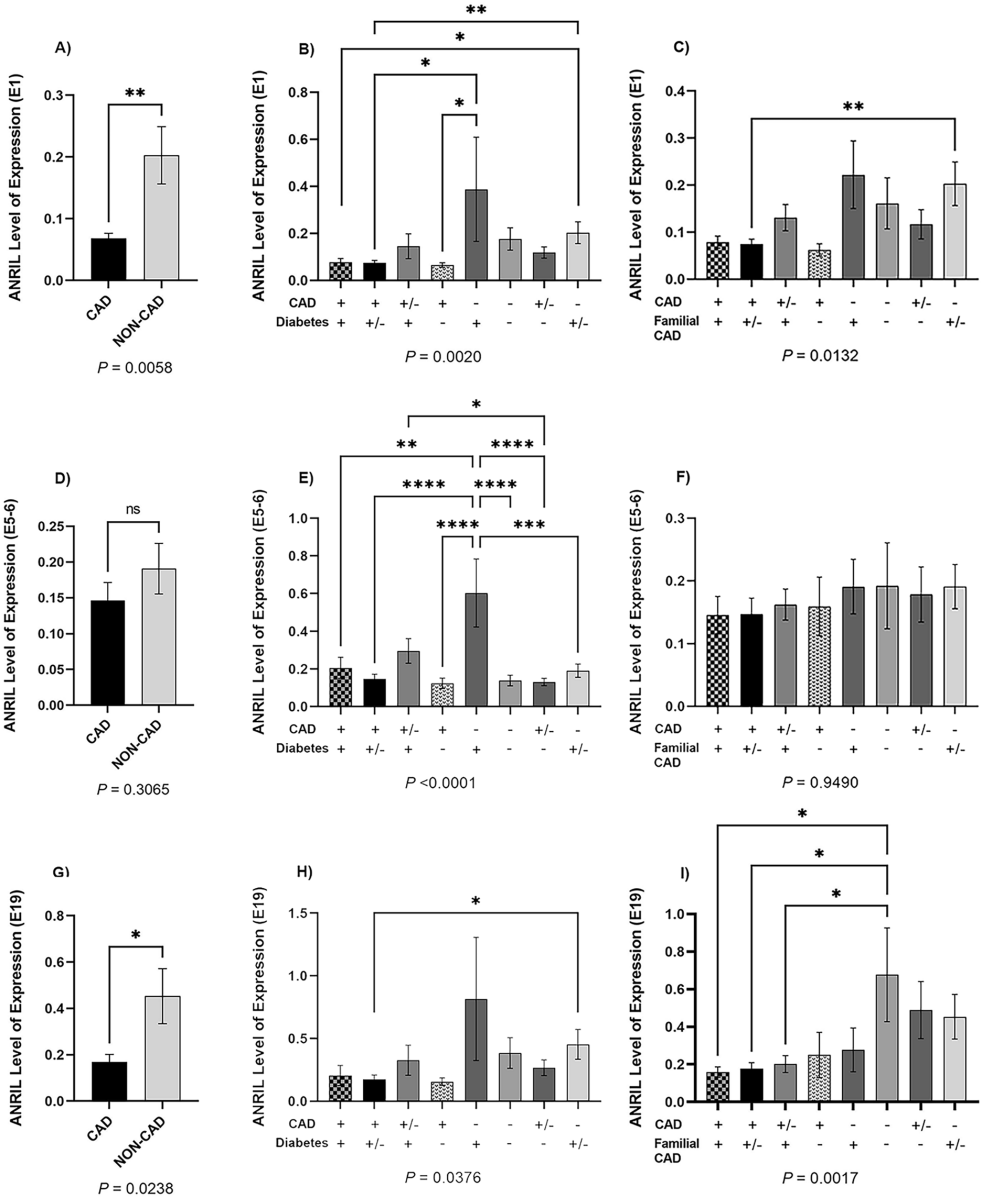
The expression of *ANRIL* regarding CAD, T2D, and the history of familial CAD. (**A**–**C**) for exon one, (**D**–**F**) for exons five-six, and G, H, and I for exon 19 detection. The expression of *ANRIL* using E1 and E19 primers was significantly lower in CAD vs non-CAD (**A**, **G**) while E5-6 primers revealed a non-significant decrease (**D**). (**B**, **E**, **H**) The expression of *ANRIL* in the presence and absence of T2D and CAD. T2D contributes to higher expression of *ANRIL*. E5-6 detection revealed a considerable downregulation of *ANRIL* in CAD patients compared to non-CAD ones and displayed a significantly higher expression of *ANRIL* in subjects with diabetes I. (**C**, **F**, **I**) The expression of *ANRIL* regarding familial CAD. E19 primers detected significant downregulation of *ANRIL* in patients with CAD as well as in those having a history of familial CAD, compared to the control group. One-way ANOVA was performed to compare expression between different categories (the respective *P*-values are indicated below each graph). Multiple comparison tests were also applied by comparing the mean of each column with the mean of every other column and were corrected by Turkey test (asterisks represent for multiple comparison tests). The presence and absence of each disease is depicted by + and − signs in front of each status in x axis (**B**, **C**, **E**, **F**, **H**, **I**). *P* values ≤ 0.0001 are given ****, while *P* ≤ 0.001, *P* ≤ 0.01, and *P* ≤ 0.05 are given ***, **, and * respectively. *ANRIL*, antisense noncoding RNA in the *INK4A* locus; CAD, Coronary Artery Disease; T2D, Type 2 Diabetes.

*ANRIL* featured 14 splicing variants in the Ref Seq database and encompassed many regulatory elements along its sequence (Fig. 1). *ANRIL* was significantly downregulated in the serum samples of the premature CAD group compared with the non-CAD control group (*P* < 0.05) (Fig. 2). The detection of *ANRIL* yielded diverse results through different primer sets. The expression analysis of *ANRIL* was performed using three different sets of primers amplifying exons 1, 5–6, and 19. The detection of middle exons (exons five-six) showed a nonsignificant decrease in *ANRIL* expression in the premature CAD group compared with the non-CAD group (Fig. 2D), while the detection of terminal exons manifested a significant downregulation (Fig. 2A,G).

### Diabetes correlated with higher *ANRIL* expression levels

Patients with both premature CAD and T2D showed lower expression of *ANRIL* than those categorized as non-CAD. Variations in *ANRIL* expression using different primers were adopted here as well (Fig. 2B,E,H). Diabetic patients with no symptoms of premature CAD indicated higher levels of *ANRIL* than those who did not have T2D or had premature CAD. Nevertheless, in the patients with premature CAD and T2D, the expression of *ANRIL* remained low altogether.

### The history of familial CAD was in line with lower *ANRIL* expression levels

A history of familial CAD per se lacked contribution to significantly lower *ANRIL* expression. However, along with premature CAD, it preserved the low expression of *ANRIL* altogether.

### Genetic variants within and proximal to the *ANRIL* sequence displayed different genotype and allele frequencies but not a significant association with premature CAD

Associations between premature CAD status and the genotypes of six SNPs were investigated. No significant associations were found between premature CAD and rs10757274, rs2383206, rs2383207, rs496892, rs10757278, and rs10738605. An overview of the studied variants is summarized in S3 Table. The odds ratios and *P* values for the different genetic models are listed in Table 2. The distributions of the genotypes for all 6 SNPs were consistent with the Hardy Weinberg law at a significance level of 0.05 (Table 2). The frequencies of risk genotypes for all the SNPs were higher in the premature CAD group than in the non-CAD group but the differences lacked statistical significance.

**Table 2:** The distribution of alleles and genotypes of variants.

	Non-CAD (N/%)	CAD (N/%)	OR (95% CI)	*P*-value
rs10757274 (A>G)				
Allele frequency				
A	62 (40.79)	72 (39.13)	1.00	0.75
G	90 (59.21)	112 (60.78)	0.93 (0.60–1.43)	
Genotypes (Codominant)				
A/A	15 (19.74)	18 (19.57)	1.00	
A/G	32 (42.10)	36 (39.13)	0.93 (0.40–2.11)	0.87
G/G	29 (38.16)	38 (41.30)	1.09 (0.46–2.48)	0.83
Genotypes (Dominant)				
A/A	15 (19.74)	18 (19.57)	1.00	
A/G–G/G	61 (80.26)	74 (80.43)	1.01 (0.47–2.16)	0.97
Genotypes (Recessive)				
A/A–A/G	47 (61.84)	54 (58.70)	1.00	
G/G	29 (38.16)	38 (41.30)	1.14 (0.62–2.10)	0.67
Genotypes (Over-dominant)				
A/A–G/G	44 (57.89)	56 (60.87)	1.00	
A/G	32 (42.11)	36 (39.13)	0.88 (0.48–1.61)	0.70
HWE†	*X*^*2*^ = 1.25 *P* = 0.53	*X*^*2*^ = 2.93 *P* = 0.23		
rs2383206 (A>G)				
Allele frequency				
A	52 (34.21)	66 (35.87)	1.00	0.75
G	100 (65.72)	118 (64.13)	0.93 (0.59–1.45)	
Genotypes (Codominant)				
A/A	9 (11.84)	13 (14.13)	1.00	
A/G	34 (44.73)	40 (43.47)	0.81 (0.32–2.11)	0.68
G/G	33 (43.42)	39 (42.39)	0.82 (0.32–2.13)	0.68
Genotypes (Dominant)				
A/A	9 (11.84)	13 (14.13)	1.00	
A/G–G/G	67 (88.16)	79 (85.87)	0.82 (0.34–1.93)	0.66
Genotypes (Recessive)				
A/A–A/G	43 (56.58)	53 (57.61)	1.00	
G/G	33 (43.42)	39 (42.39)	0.96 (0.53–1.73)	0.89
Genotypes (Over-dominant)				
A/A–G/G	42 (55.26)	52 (56.52)	1.00	
A/G	34 (44.73)	40 (43.47)	0.95 (0.52–1.71)	0.87
HWE	*X*^*2*^ = 0.002 *P* = 0.99	*X*^*2*^ = 0.28 *P* = 0.87		
rs2383207 (A>G)				
Allele frequency				
A	51 (34)	63 (34.24)	1.00	0.96
G	99 (66)	121 (65.76)	0.98 (0.62–1.56)	
Genotypes (Codominant)				
A/A	8 (10.66)	12 (13.04)	1.00	
A/G	35 (46.66)	39 (42.39)	0.74 (0.26–2.07)	0.56
G/G	32 (42.66)	41 (44.56)	0.85 (0.30–2.40)	0.76
Genotypes (Dominant)				
A/A	8 (10.66)	12 (13.04)	1.00	
A/G–G/G	67 (89.33)	80 (86.96)	0.79 (0.31–2.00)	0.64
Genotypes (Recessive)				
A/A–A/G	43 (57.33)	51 (55.43)	1.00	
G/G	32 (42.66)	41 (44.56)	1.08 (0.59–1.96)	0.80
Genotypes (Over-dominant)				
A/A–G/G	40 (53.33)	53 (57.61)	1.00	
A/G	35 (46.67)	39 (42.39)	0.84 (0.46–1.52)	0.58
HWE	*X*^*2*^ = 0.11 *P* = 0.94	*X*^*2*^ = 0.31 *P* = 0.85		
rs496892 (C>T)				
Allele frequency				
C	108 (71.05)	124 (68.89)	1.00	0.66
T	44 (28.95)	56 (31.11)	1.10 (0.69–1.77)	
Genotypes (Codominant)				
C/C	38 (50)	45 (50)	1.00	
C/T	32 (42.10)	34 (37.77)	0.89 (0.46–1.73)	0.74
T/T	6 (7.89)	11 (12.22)	1.54 (0.56–4.69)	0.42
Genotypes (Dominant)				
C/C	38 (50)	45 (50)	1.00	
C/T–T/T	38 (50)	45 (50)	1.00 (0.54–1.82)	> 0.99
Genotypes (Recessive)				
C/C–C/T	70 (92.11)	79 (87.78)	1.00	
T/T	6 (7.89)	11 (12.22)	1.62 (0.55–4.62)	0.35
Genotypes (Over-dominant)				
C/C–T/T	44 (57.89)	56 (62.22)	1.00	
C/T	32 (42.10)	34 (37.77)	0.83 (0.45–1.53)	0.57
HWE	*X*^*2*^ = 0.04 *P* = 0.97	*X*^*2*^ = 1.26 *P* = 0.53		
rs10757278 (A>G)				
Allele frequency				
A	67 (45.27)	79 (43.41)	1.00	0.73
G	81 (54.73)	103 (56.59)	1.07 (0.68–1.68)	
Genotypes (Codominant)				
A/A	17 (22.97)	21 (23.07)	1.00	
A/G	33 (44.59)	37 (40.65)	0.90 (0.42–2.05)	0.81
G/G	24 (32.43)	33 (36.26)	1.11 (0.49–2.48)	0.79
Genotypes (Dominant)				
A/A	17 (22.97)	21 (23.07)	1.00	
A/G–G/G	57 (77.03)	70 (76.92)	0.99 (0.48–2.02)	0.98
Genotypes (Recessive)				
A/A–A/G	50 (67.57)	58 (63.74)	1.00	
G/G	24 (32.43)	33 (36.26)	1.18 (0.62–2.23)	0.60
Genotypes (Over-dominant)				
A/A–G/G	41 (55.41)	54 (59.34)	1.00	
A/G	33 (44.59)	37 (40.65)	0.85 (0.46–1.55)	0.61
HWE	*X*^*2*^ = 0.74 *P* = 0.69	*X*^*2*^ = 2.70 *P* = 0.25		
rs10738605 (C>G)				
Allele frequency				
C	46 (31.08)	59 (33.15)	1.00	0.69
G	102 (68.92)	119 (66.85)	0.90 (0.57–1.47)	
Genotypes (Codominant)				
C/C	6 (8.10)	13 (14.60)	1.00	
C/G	34 (45.94)	33 (37.07)	0.44 (0.14–1.36)	0.13
G/G	34 (45.94)	43 (48.31)	0.58 (0.19–1.73)	0.31
Genotypes (Dominant)				
C/C	6 (8.10)	13 (14.60)	1.00	
C/G–G/G	68 (91.89)	76 (85.39)	0.51 (0.18–1.38)	0.19
Genotypes (Recessive)				
C/C–C/G	40 (54.05)	46 (51.69)	1.00	
G/G	34 (45.94)	43 (48.31)	1.10 (0.60–2.00)	0.76
Genotypes (Over-dominant)				
C/C–G/G	40 (54.05)	56 (62.92)	1.00	
C/G	34 (45.94)	33 (37.07)	0.69 (0.37–1.28)	0.25
HWE	*X*^*2*^ = 0.38 *P* = 0.82	*X*^*2*^ = 2.37 *P* = 0.30		

The frequency of alleles and genotypes of rs10757274, rs2383206, rs2383207, rs496892, rs10757278, and rs10738605 in CAD and non-CAD groups are listed. The odd ratios and *P-*values are outputs of χ^2^ tests. For Hardy–Weinberg Equilibrium, χ^2^ values are also included in the table.

**†**Hardy–Weinberg Equilibrium.

### An association existed between *ANRIL* expression and the genotypes of rs10757274, rs2383206, rs2383207, rs496892, rs10757278, and rs10738605

While rs10738605 resided in exon six of *ANRIL*, the other SNPs were in noncoding regions. Among these, rs10757274 and rs496892 are included in the sequences of long interspersed elements (LINEs). Multiple binding sites for transcription factors (TFs) were included in the regions containing the studied SNPs (S2 Fig.).

**Figure 3:**
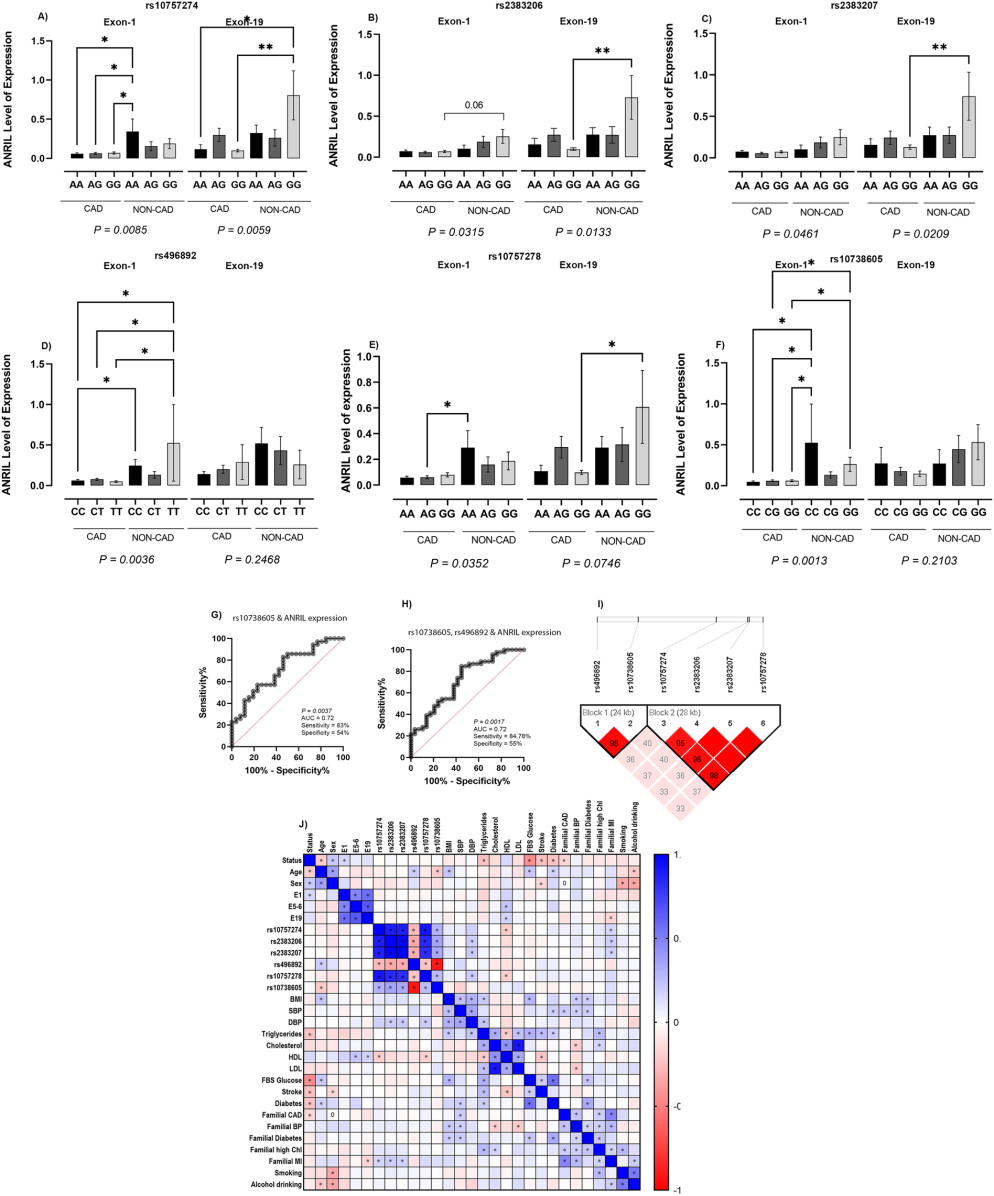
The correlation between SNPs within and proximal to the *ANRIL* sequence and the serum expression of *ANRIL* (both exon one and exon 19) in premature CAD compared to the non-CAD group. The risk genotype for rs10757274: A>G, rs2383206: A>G, rs2383207: A>G, rs10757278: A>G and rs10738605: C>G is GG, and the risk genotype for rs496892: C>T is TT (**A**–**F**). The P- values are regarding one-way ANOVA analyses between all groups and asterisks represent the P-values < 0.05 in multiple comparison analyses. (**G**, **H**) ROC curve analysis for rs10738605, rs496892, and the expression of *ANRIL*. (**I**) Linkage disequilibrium analysis for the selected SNPs. The name of studied SNPs are at the top of the figure and D’ factor (%) is displayed in the squares below. The empty red squares indicate Dʹ > 0.9. rs10757274, rs2383206, rs2383207 and rs10757278 are in one block and rs496892 and rs10738605 share a block together. (**J**) The Spearman correlation between all variables. The graph is generated using the Spearman correlation coefficients (r). The scale bar on the right demonstrates the range of the r coefficient in color. The r values close to 1 (blue squares) demonstrate an increasing monotonic pattern between variables and values close to − 1 (red squares) correspond to decreasing monotonic trends between variables. P values ≤ 0.0001 are given ****, while P ≤ 0.001, P ≤ 0.01, and P ≤ 0.05 are given ***, **, and * respectively. *ANRIL* antisense noncoding RNA in the *INK4A* locus, *CAD* Coronary Artery Disease, *ROC* Receiver Operating Characteristic, *BMI* body mass index, *DBP* diastolic blood pressure, *FBS* fasting blood sugar, *HDL-C* high-density lipoprotein-cholesterol, *LDL-C* low-density lipoprotein-cholesterol, *SBP* systolic blood pressure, *T2D* Type 2 diabetes, *TC* total cholesterol.

The expression of *ANRIL* in the premature CAD subjects carrying the risk genotypes was lower than that in the control group (Fig. 3). For rs10757274, rs2383206, rs2383207, and rs10757278, the expression of exon 19 of *ANRIL* (a representative of long isoforms) was significantly downregulated in PCAD carriers of risk genotypes, however, detection of the first exon of *ANRIL* was significantly reduced in PCAD carriers of risk genotypes by rs496892 and rs10738605. For rs10757274, rs2383206, rs2383207, rs10757278, and rs10738605, the risk genotypes were GG compared to TT for rs496892.

### The Receiver Operating Characteristic (ROC) analysis revealed that *ANRIL* expression could differentiate premature CAD from non-CAD regarding rs10738605 and rs496892

The ROC analysis was performed to investigate whether subjects with the risk genotypes of the studied SNPs could be differentiated into premature CAD and non-CAD groups regarding *ANRIL* expression. The ROC curves (Fig. 3G,H) revealed that in the subjects carrying the risk genotype of rs10738605, the expression of *ANRIL* was capable of separating premature CAD from non-CAD subjects with a sensitivity of 83%. Further, in the subjects carrying the risk genotypes for a combination of rs10738605 and rs496892, *ANRIL* expression predicted the patients correctly with 84% sensitivity (Fig. 3H).

### The LD analysis ascertained two distinct LD blocks

Haploview revealed strong LD between rs10757274, rs2383206, rs2383207, and rs10757278 and between rs496892 and rs10738605. Haploview demonstrated two distinct blocks: one for rs496894 and rs10738605 and another block for rs10757274, rs2383206, rs2383207, and rs10757278 (Fig. 3I).

### The correlation analysis demonstrated strong links between some variables

The correlation matrix is depicted as a heat map in Fig. 3J. A negative correlation was detected between the status of premature CAD and age, triglyceride, fasting blood sugar, stroke history, T2D, and familial CAD. Positive correlations were observed between the expression of *ANRIL* using different primers (E1, E5-6, and E19), and between the status of the rs10757274, rs2383206, rs2383207, and rs10757278 genotypes as well. Some weaker positive correlations were discovered between the expression of *ANRIL* and the level of high-density lipoprotein and between familial CAD and familial myocardial infarction. Positive correlations were also indicated between the levels of triglyceride, cholesterol, fasting blood sugar, stroke history, and T2D (Fig. 3J).

## Discussion

Atherosclerosis normally progresses in the form of late plaques in the fourth decade of life and involves older people. Nonetheless, in some cases, women aged below 55 and men younger than 45 experience CAD symptoms^3^. The principal cause of such incidence remains indefinite, although previous studies have reported the contribution of genetic factors in promoting atherosclerosis at younger ages^6,24^.

The gold-standard diagnosis of CAD is angiography, which is an invasive technique. Developing noninvasive and low-cost screening methods to predict premature CAD occurrence in young adults is crucial since CAD has various socioeconomic effects on populations.

According to a previous study, *ANRIL* expression exhibited post-treatment recovery. A negative correlation also existed between the number of diseased vessels and the expression of *ANRIL*^11^. The detection of terminal exons (E1 and E19) manifested promising potential to differentiate premature CAD from non-CAD, while the identification of medial exons (E5-6) lacked such a capability. This might be due to the differential expression of linear and circular isoforms in serum since internal exons are mostly enriched in circular variants^10^.

Diabetes is the most common risk factor for premature CAD in the Iranian population^2^. It is associated with higher levels of *ANRIL* expression. Our CAD patients who suffered from T2D demonstrated lower expression of *ANRIL*, denoting the dominant effect of CAD over T2D. In contrast, our diabetic patients with no premature CAD symptoms expressed *ANRIL* to significantly higher extents. Our data chime in with the findings of a previous study^25^. A history of familial CAD was in keeping with the influence of CAD itself on the expression of *ANRIL* and demonstrated no independent association with CAD.

A substantial body of evidence indicates that lncRNAs function through their distinct secondary structure, resulting in the dynamic accessibility of exons during cellular functions. The results of a study demonstrated a relationship between the presence of Alu elements in exons and their differential expression^10^.

There are shreds of evidence that noncoding variants can affect expression as well as exonic ones. Intronic SNPs exert their functional role through their existence in exon–intron junctions, splicing branch points and enhancers, or through the creation of cryptic splice sites. Although most functional SNPs reside within a 30-bp distance from splice sites, SNPs in the middle of introns should not be underestimated^26^.

While some of our investigated SNPs reside in the binding sites of transcription factors, some are included in LINEs, explaining the regulatory effect of these variants on *ANRIL* expression. LINEs can regulate gene expression by mediating chromatin remodeling, regulating transcription, and altering the stability of transcripts. They can function as promoters or enhancers as well^27^.

Previous studies on the Iranian population have introduced rs10757274 and rs2383206 as SNPs associated with CAD^28–30^. Only a few investigations are available on premature CAD in the Iranian population^2^.

There are few studies on *ANRIL* expression in the Iranian population. Previous studies in the Iranian population measured *ANRIL* expression in peripheral blood samples from patients with (aged) CAD^25,31^, whereas this study examined *ANRIL* expression in sera of premature CAD patients.

Serum provides a higher sensitivity for biomarker detection and contains stable RNAs mostly enriched in extracellular vesicles^20^. As of yet, there are no serum expression data available for the Iranian population. Yari et al.^31^ demonstrated no significant difference in the expression of NR_003529 transcript between CAD patients and the control group. However, we revealed a significant association between lower expression of *ANRIL* (NR_003529), the longest *ANRIL* transcript, and premature CAD in the Iranian population. Despite this discrepancy, their expression analysis of EU741058 transcript was consistent with ours regarding the first exon primer. Rahimi et al.^25^ reported an upregulation in *ANRIL* expression in diabetic patients with CAD compared to non-CAD diabetic subjects. Here, we investigated *ANRIL* expression in premature CAD patients with and without diabetes. Serum expression of *ANRIL* showed a different pattern compared to peripheral blood mononuclear cells' expression.

The correlation between *ANRIL* expression and the status of SNPs in its sequence has not yet been reported in Iranian patients with premature CAD. Here, we demonstrated that PCAD carriers of risk genotypes for rs496892 and rs10738605 showed lower expression of first exon of *ANRIL*. rs496892 and rs10738605, both are proximal to the first exons of *ANRIL* and revealed a strong linkage disequilibrium in our population. On the contrary, rs10757274, rs2383206, rs2383207, and rs10757278 reside in the 3' of *ANRIL* and correlate with lower expression of exon 19. These four SNPs share a haplotype block together supporting our findings on the effect of these SNPs on expression of terminal exons of *ANRIL*.

Linking DNA variants to function is one of the major challenges in deciphering the influence of genetics on CAD. Unlike monogenic diseases, multifactorial disorders result from many small contributing factors. Therefore, large-scale studies are needed to validate the association between these variants and diseases^32^.

Our ROC analysis demonstrated that *ANRIL* lacked predictive value as a biomarker for premature CAD per se (data not shown). Nevertheless, *ANRIL* expression could distinguish between premature CAD and non-CAD in carriers of rs496892 and rs10738605 risk genotypes (Fig. 3G,H). In this group, the expression of *ANRIL* could accurately determine the status of premature CAD by 83% and 84% sensitivity (respectively for rs10738605 and a combination of rs10738605 and rs496892); still, the specificity values were slight (54% and 55%, respectively). Further research requires larger sample sizes.

GWASs are incapable of discovering disease-causing SNPs solely. They only narrow down genetic variants to the most associated SNPs with the disease. Due to the population stratification in large-scale GWASs, the confirmation of the associations by replication studies in smaller and more homogenous populations is crucial^33,34^. Our findings did not replicate the association between the studied SNPs and premature CAD significantly. It might be a consequence of the small sample size, which was limited because of the exclusive age group of the included subjects. As proposed by the 1000 Genome Project, genetic variants are categorized into four groups: population-specific, continental area-specific, continental areas-shared, and all continents-shared^35^. The structure of the population also contributes to differences in the frequency of genotypes between sub-populations even in a shared geographic location^36^. It is essential to investigate the association between genetic variants and diseases in populations of various ancestries. Different expression and LD profiling among populations were convincing evidence to discover the expression, LD, and genetic association screening of the *ANRIL* region in the Iranian population. The interplay of SNP and expression has been reported to vary between populations as well^37–39^. The association between genetic variants and diseases in 1 population does not simply generalize to other populations. Besides ethnicity, age and other environmental factors affect the variation^40^.

DNA variants and RNA expression lack predictive value when used solely, while a combination of both approaches is worthwhile. The last decade has witnessed a considerable fall in the costs of sequencing and expression analyses, raising the hopes of using genetics as a helping arm in clinics. Our principal notion is that a combination of DNA and RNA variants is capable of predicting premature CAD in young adults. Premature CAD is considered a burden on the health systems of countries: not only does it negatively impact a young workforce but also it contributes to societal challenges. Traditional screening methods lack the strength to precisely predict premature CAD^41^. What could substantially strengthen the armamentarium is the recruitment of artificial intelligence and machine learning.

## Limitations of this study

It is necessary to consider the limitations of the current study when interpreting its outcomes. Firstly, our small sample size precludes the generalizability of our results concerning the association of rs10757274, rs2383206, rs2383207, rs496892, rs10757278, and rs10738605 in Iranian patients with premature CAD. Secondly, our age threshold limited the number of participants in this study. Therefore, we strongly recommend replicating this study with a larger sample size of the Iranian population. To gain a better understanding of the independent effect of premature CAD occurrence on *ANRIL* expression, parallel studies on older individuals with CAD are strongly recommended.

## Methods

### Sample collection

The present study recruited 93 premature CAD and 87 non-CAD age-matched subjects hospitalized at Rajaie Cardiovascular Medical and Research Center, Tehran, Iran, between 2017 and 2019. All patients were diagnosed by angiography. A questionnaire and a formal consent form were completed and signed by all the subjects. The study protocol was approved by the Ethics Committee of Rajaie Cardiovascular Medical and Research Center (RHC.AC.IR.REC.1396.62), and was conducted in accordance with the Helsinki Declaration. The informed consent was obtained from all subjects and/or their legal guardian(s). Patients (women < 55 y/o and men < 49 y/o) diagnosed with premature CAD by coronary angiography were included, while patients with malignant tumors, severe liver disease, severe kidney dysfunction, infectious diseases, immune diseases, communication dysfunction, or cognitive dysfunction were excluded. The non-CAD group included women < 55 y/o and men < 49 y/o with no evidence of coronary artery disease. The exclusion criteria was as well as the one applied for CAD subjects. T2D patients with and without PCAD were also included in the study. All patients in Rajaie Cardiovascular Medical and Research Center were evaluated by the medical team for their diabetes status before being included in the current study. Clinical considerations regarding diabetes have been taken into account by clinicians prior to any treatment or intervention.

### DNA extraction

DNA was extracted from peripheral blood using the DNeasy Blood & Tissue Kit (QIAGEN, Germany). The quantity of the extracted DNAs was assessed using NanoDrop 2000/2000c Spectrophotometers (Thermo Fisher Scientific, USA).

### Serum isolation, RNA extraction, and cDNA synthesis

Following blood collection, serum was isolated by incubation at room temperature for 30 min. Afterward, clots were removed by centrifuge at 3500×*g* for 20 min. Subsequently, the serum was isolated in a ribonuclease (RNase)-free tube and kept at − 80 ℃ for longer preservation.

Total RNAs were isolated from serum using a plasma/ serum RNA purification kit (NORGEN BIOTEK, Canada). The RNAs were then concentrated by the RNA Clean-up and Concentration Kit (NORGEN BIOTEK, Canada). Subsequently, reverse transcription was performed with a PrimeScript First-Strand cDNA Synthesis Kit (Takara, Japan).

### Real-time polymerase chain reaction (PCR)

Specific PCR primers were designed using Gene Runner (version 6.5.51) and Primer3, synthesized by Macrogen (Korea) (S1 Table). The primers were designed for different exons of *ANRIL* (RefSeq NR_003529.3): one pair on exon one, one on exon five-six, and one on exon 19 (Fig. 1). The real-time PCR analysis was performed using the BioFACT 2X Real-Time PCR Master Mix (High ROX), including SYBR Green (BioFACT, South Korea), in an Applied Biosystems StepOne Plus instrument (Applied Biosystems, USA). Next, cDNA was added to 10 μL of SYBR Green and 0.5 μM of each primer in a 20 μL reaction. All the reactions were repeated in duplicates, and mean threshold cycles were used for further analyses. The real-time thermal program was as follows: 95 °C for 30 s and 62 °C for 35 s, for which *5srRNA* was utilized as an internal control. The expression level was calculated by 2^−ΔCt^.

### PCR and sequencing

For SNP genotyping, primers were designed to detect a 300–700 base pair (bp) product. The amplicon was then sequenced. The sequence and characteristics of the primers used in this study are listed in S1 Table. Afterward, 100 ng DNA was added to 10 μL of Taq DNA Polymerase Master Mix RED (Ampliqon, Denmark) for PCR reactions, along with 0.5 μM of each primer in each 20 μL reaction. PCR was performed for 30 cycles in the following thermal program: 95 °C for 5 min, 30 cycles at 95 °C for 30 s, 61 °C for 30 s, 72 °C for 30 s, and a final extension at 72 °C for 5 min. The PCR products were electrophoresed on a 1–2% agarose gel, stained with FluoroDye (SMOBIO, Taiwan), and visualized under ultraviolet light. Subsequently, Sanger sequencing was performed, and the sequencing files were analyzed using CodonCode Aligner 10.0.2 for Windows (www.codoncode.com).

### Linkage disequilibrium (LD) analysis

Haploview (Haploview Software, USA, www.broadinstitute.org/haploview) was employed to analyze LD^42^. Haploview generated D’, logarithm of the odds (LOD), and r^2^ factors. The results are presented in S2 Table.

### In silico analysis

#### Finding transcription-factor binding sites in the SNPs’ regions

Transcription-factor (TF) binding sites in *ANRIL* introns were discovered through the application of related databases in the UCSC Genome Browser track data hubs (https://genome.ucsc.edu/)^43^. The list of the databases is as follows: TF ChIP-seq Clusters from ENCODE3, eCLIP (by biosample) (ENCODE), eCLIP (by target) (ENCODE), ENCODE TFs, ENC RNA Binding, ENC TF Binding, TFBS Conserved, JASPAR CORE 2022-Predicted TF Binding Site, TOBIAS footprint prediction, human P53 Binding And Expression Resource (BAER) hub, ReMap 2022 Regulatory Atlas, and UniBind 2021 Hub.

### Statistical analysis

The mean and the standard deviation were calculated in GraphPad Prism 9 for Windows (GraphPad Software, San Diego, California USA, www.graphpad.com) for each parameter between the premature CAD and non-CAD subjects. Risk-factor probabilities were analyzed with the aid of the χ^2^ test for qualitative variables and the *t* test for quantitative variables. The D'Agostino–Pearson omnibus normality test was also performed. Additionally, a nonparametric *t* test (Mann–Whitney) was applied for values not fitting in a normal distribution. One-way ANOVA was utilized to analyze the values of more than two groups, and the Spearman correlation was applied to generate a correlation matrix for all variables. The Receiver Operating Characteristic (ROC) analysis was applied to assess the diagnostic potential of *ANRIL* expression. ROC curves were drawn using GraphPad Prism 9.

### Ethics approval

The study protocol was approved by the Ethics Committee of Rajaie Cardiovascular Medical and Research Center (RHC.AC.IR.REC.1396.62), and the study was conducted in accordance with the Helsinki Declaration. The informed consent was obtained from all subjects and/or their legal guardian(s).

### Supplementary Information

Supplementary Information.

## Data Availability

The datasets generated during and/or analysed during the current study are available in the GenBank repository, sequences are accessible via OQ744705–OQ745198 accession numbers for CAD patients and OQ745199–OQ745577 for non-CAD subjects. The detailed list of accession numbers is available from the corresponding author.
