# Supplementary material for: Associations between low serum levels of ANRIL and some common gene SNPs in Iranian patients with premature coronary artery disease
Source: Sci Rep. 2024 Jan 13;14:1244. doi: 10.1038/s41598-024-51715-2 (PMC10787829; doi:10.1038/s41598-024-51715-2)
Supplement: Supplementary file 1 — Supplementary Information. [file 41598_2024_51715_MOESM1_ESM.pdf]

Associations between low serum levels of ANRIL and some common gene SNPs in patients with premature CAD

Elham Taheri Bajgan[1], Ali Zahedmehr[2], Farshad Shakerian[2,3], Majid Maleki[3], Hooman Bakhshandeh[3], Seyed Javad Mowla[1]*, Mahshid Malakootian[3]*

1- Molecular Genetics Department, Faculty of Biological Sciences, Tarbiat Modares University, Tehran, Iran
2- Cardiovascular Intervention Research Center, Rajaie Cardiovascular Medical and Research Center, Iran University of Medical Sciences, Tehran, Iran
3- Cardiogenetics Research Center, Rajaie Cardiovascular Medical and Research Center, Iran University of Medical Sciences, Tehran, Iran

* Corresponding Authors:
malakootian@rhc.ac.ir (MM)
sjmowla@yahoo.com (SJM)

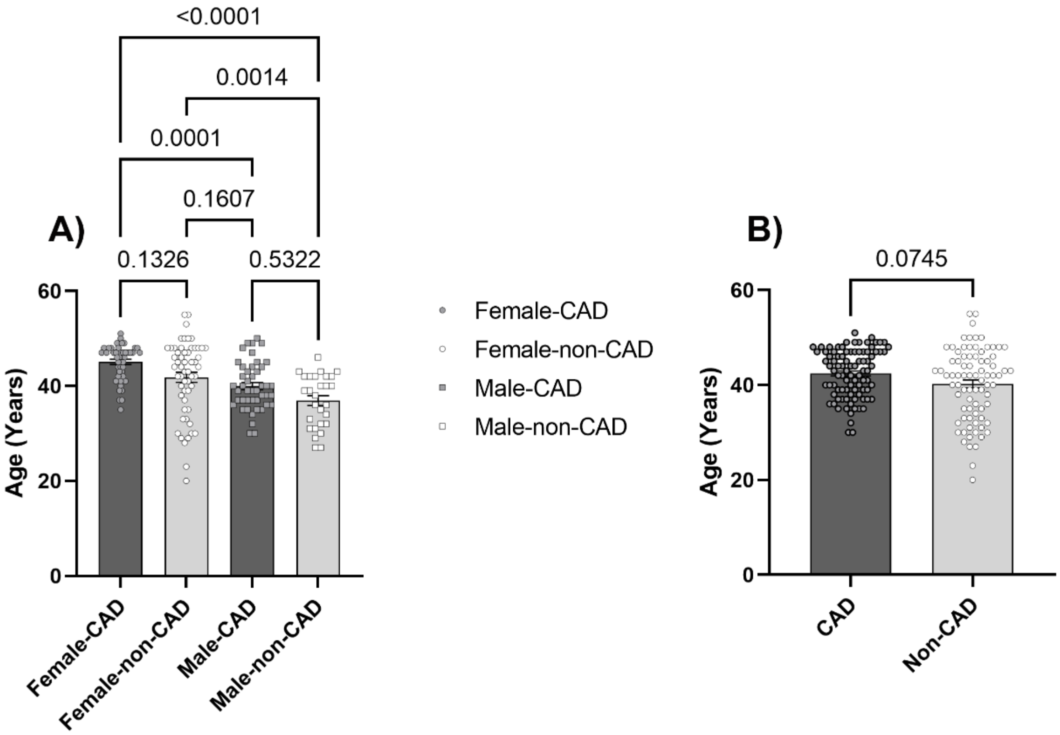

S1 Figure. The age distribution graphs respective (A) and irrespective (B) to sex. A) non-parametric one-way ANOVA was performed to compare the difference between the four groups. *P*-values are demonstrated above each comparison. *P*-values are retrieved from multiple comparison analysis in GraphPad prism. B) t-test was used to compare the difference between age in CAD and non-CAD groups irrespective to sex. *P*-value is indicated above the comparison line.

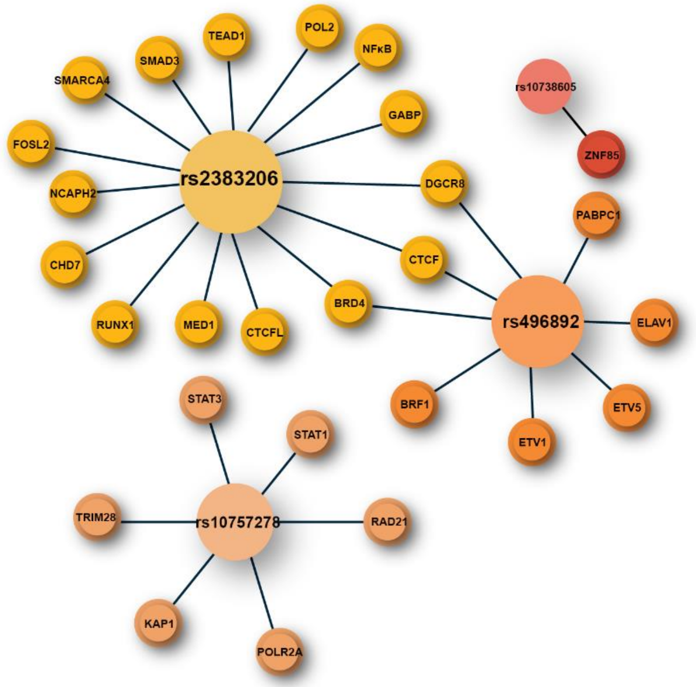

S2 Figure. The *in silico* analysis. The network of SNPs and the transcription factors that their binding sites include the studied SNPs. The list of transcription factors is as follows: rs2383206 for DGCR8, CTCF, GABP, NFκB, Pol2, CTCFL, MED1, RUNX1, BRD4, CHD7, NCAPH2, FOSL2, and TEAD1; rs496892 for DGCR8, CTCF, PABPC1, ELAV1, ETV5, ETV1, BRD4, and BRF1; rs10757278 for STAT1, STAT3, POLR2A, RAD21, KAP1, and TRIM28; and rs10738605 for ZNF85.

S1 Table. The sequence and features of primers.

Number	Name	Gene / Transcript	Exon / Intron number	Forward (F)/ Reverse (R)	Sequence	Product Length (bp)
1	rs10757274	CDKN2B-AS1 (ANRIL)	Intron 12	F	GTGATGGGAGGTACTGGTATTAC	378
				R	GAACAATGCCAAGTTCATAGTCAGG	
2	rs2383206	CDKN2B-AS1	Intron 16	F	CAGAGGCAGCGGCTAAGACCAT	438
				R	GGAGTCCAAACATTGGTACATAC	
3	rs2383207	CDKN2B-AS1	Intron 16	F	TGGACTTGGGGATTATTGGTGG	479
				R	CAGACTACCTTGTGTCTCATCACAC	
4	rs496892	CDKN2B-AS1	Intron 1	F	CCCACAAAAATGCCTTGCCTTCAC	689
				R	GTCCGTATCAAAAGCAAACCTAC	
5	rs10757278	CDKN2B-AS1	Intergenic region	F	CACAATCCCACATTTTAAGGGC	473
				R	GCTAGACTCCACGCTGTTC	
6	rs10738605	CDKN2B-AS1	Exon 6	F	TGACTAGGCTAATACACTATGTGG	309
				R	GCTTGTGTTTGGACAGAGTAGG	
7	E1	NR_003529	Exon 1	F	TACATCCGTCACCTGACACG	185
				R	TGAGATGACCTCGCTTTCCT	
8	E5-6	NR_003529	Exon 5-6	F	GCCTCATTCTGATTCAACAGCAG	159
				R	GATTCCAGCACACCTAACAGTG	
9	E19	NR_003529	Exon 19	F	TGCTTACCTAGTGCCAGATGC	150
				R	ATTACCAGCGGTGCAACTTC	
10	5s rRNA	NR_023363	Exon 1	F	GTCTACGGCCATACCACCCTG	121
				R	AAAGCCTACAGCACCCGGTAT	

All exon and intron numbers correspond to the sequence of exons and introns in transcript variant. 1 of each gene.

S2 Table. The linkage disequilibrium analysis.

D’<br>r^2<br>LOD	rs2383206	rs2383207	rs496892	rs10757278	rs10738605
rs10757274	0.95<br>0.75<br>45.31	0.96<br>0.73<br>43.76	0.37<br>0.08<br>3.5	0.98<br>0.80<br>52.21	0.40<br>0.11<br>4.49
rs2383206		1.00<br>0.95<br>63.52	0.37<br>0.11<br>4.47	1.00<br>0.67<br>41.8	0.40<br>0.14<br>5.62
rs2383207			0.33<br>0.09<br>3.74	1.00<br>0.63<br>38.8	0.36<br>0.12<br>4.83
rs496892				0.33<br>0.06<br>2.45	0.98<br>0.85<br>49.71
rs10757278					0.37<br>0.08<br>3.3

The values of D’, r2, and LOD are listed for each pair of SNPs from Haploview.

S3 Table. Variant table.

**Variant ID**	**Genomic position**	***In silico* prediction**		**ACMG classification**
		**CADD (PHRED)**	**DANN (Score)**	
rs10757274: A>G	hg.38 chr9- 22096056	4.845	Benign (0.7604)	Benign
rs2383206: A>G	hg.38 chr9-22115027	0.029	-	Benign
rs2383207: A>G	hg.38 chr9-22115960	0.034	-	Benign
rs496892: C>T	hg.38 chr9-22024352	1.407	-	Benign
rs10757278: A>G	hg.38 chr9-22124478	11.93	-	Benign
rs10738605: C>G	hg.38 chr9-22049131	4.736	-	Benign

Characteristics of the included genetic variants. The variant ID from dbSNP153, the genomic position, and in silico prediction by CADD and DANN tools are listed in the table. The last column contains the ACMG classification for each SNP.
